# Supplementary material for: Helicity: A Non-Conventional Stereogenic Element for Designing Inherently Chiral Ionic Liquids for Electrochemical Enantiodifferentiation
Source: Molecules. 2021 Jan 9;26(2):311. doi: 10.3390/molecules26020311 (PMC7828060; doi:10.3390/molecules26020311)
Supplement: Supplementary file 1 [file molecules-26-00311-s001.pdf]

Supporting Information

Helicity: a non-conventional stereogenic element for designing inherently chiral ionic liquids for electrochemical enantiodifferentiation.

Francesca Fontana*, Greta Carminati, Benedetta Bertolotti, Patrizia Romana Mussini, Serena Arnaboldi, Sara Grecchi, Roberto Cirilli, Laura Micheli and Simona Rizzo*

SI.1 Electrochemical characterizations

*Compound **1**.*

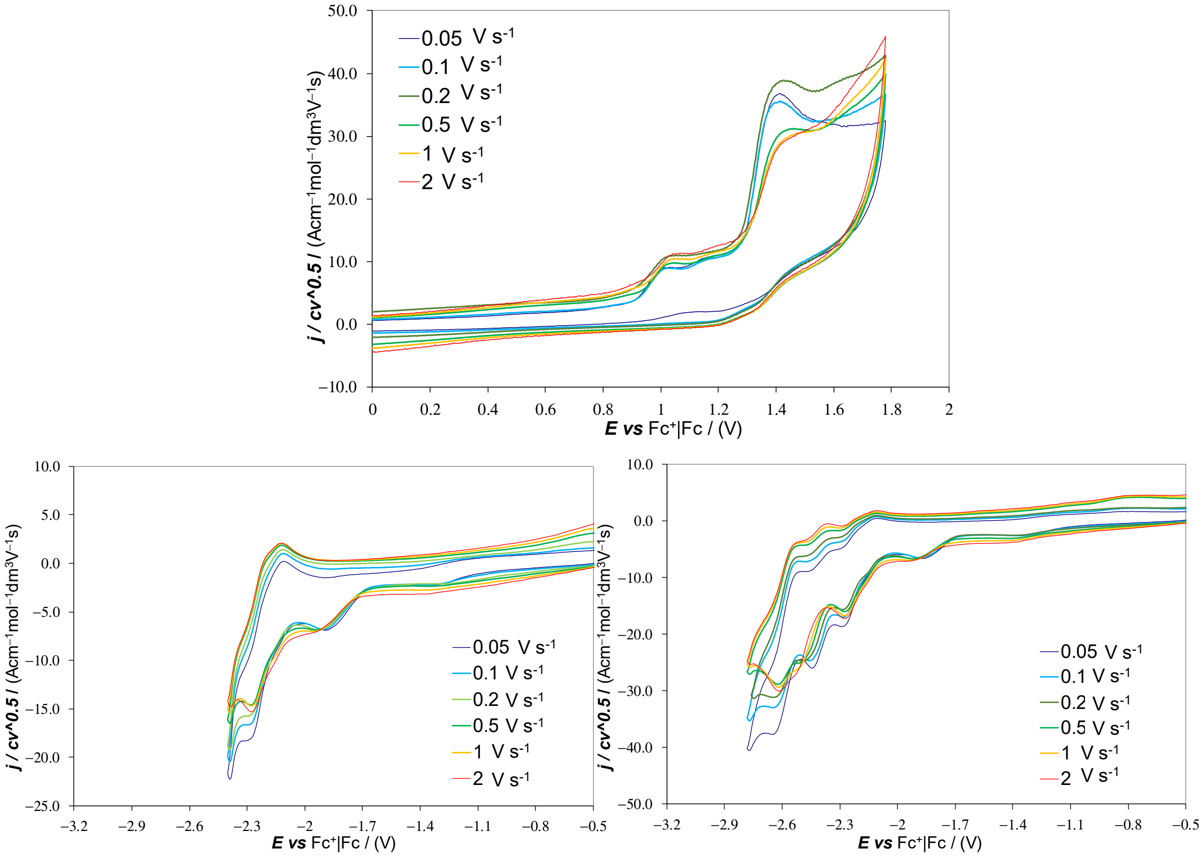

Figure S.1. Normalized CV patterns of compound **1** (0.00075 M) at scan rates in the 0.02-2 V/s range, in acetonitrile, with 0.1 M $TBAPF_6$ as supporting electrolyte, applying ohmic drop compensation by the positive feedback method and referring the potentials to the $Fc^+|Fc$ redox couple (the intersolvental standard recommended by IUPAC) measured in the same conditions (~0.39 V *vs* SCE).

*Compound **2**.*

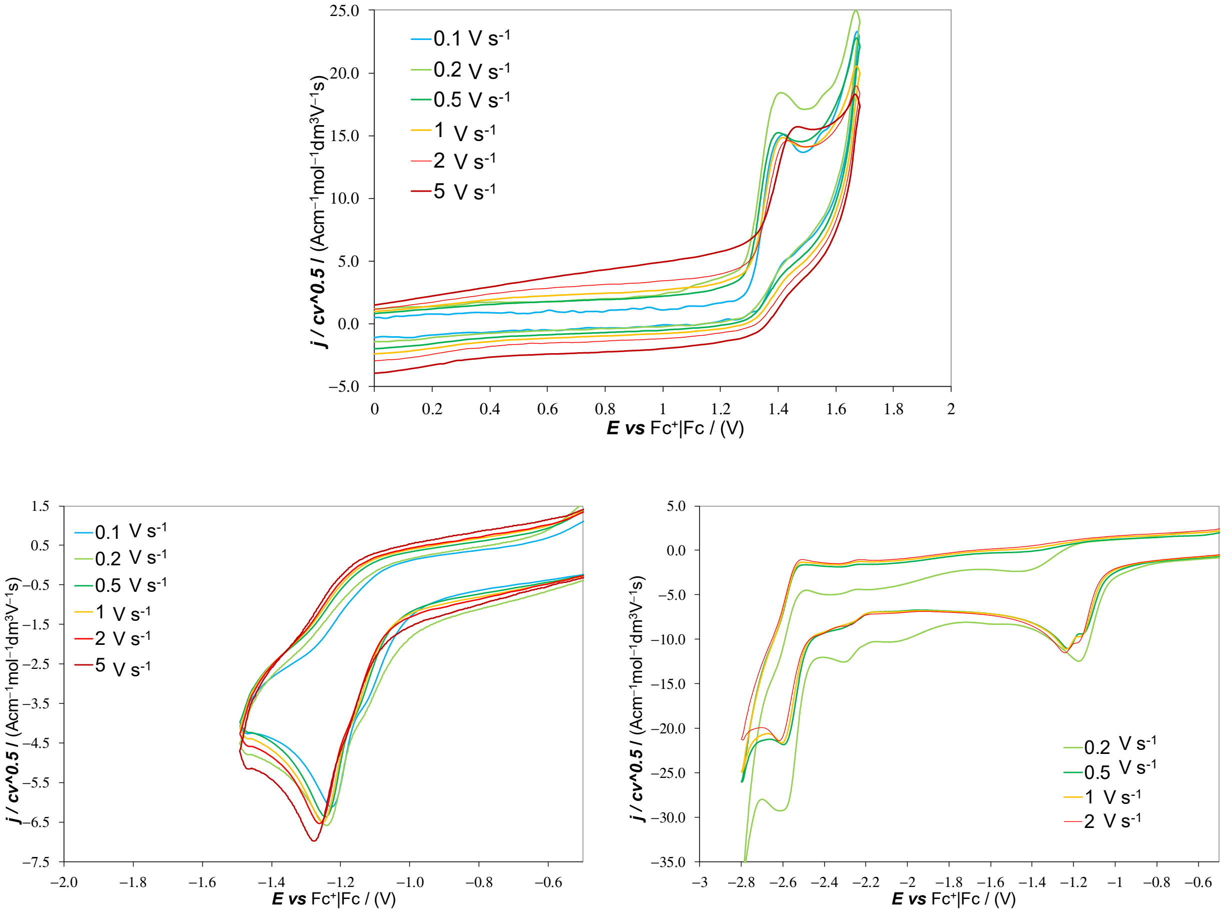

Figure S.2 Normalized CV pattern of compound **2** (0.00075 M) at scan rates in the 0.02-2 V/s range, in acetonitrile, with 0.1 M $TBAPF_6$ as supporting electrolyte, applying ohmic drop compensation by the positive feedback method and referring the potentials to the Fc^+|Fc redox couple (the intersolvental standard recommended by IUPAC) measured in the same conditions (~ 0.39 V *vs* SCE).

SI.1.1 First reduction peak of compound 2

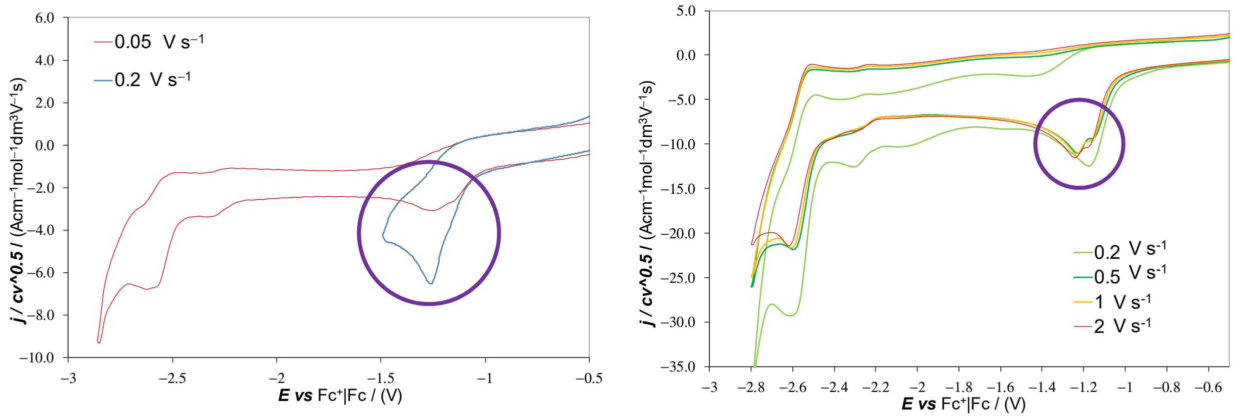

Figure S.3. The first reduction peak of **2** tending to split or to feature a preceding shoulder, confirmed in two experiments at different times and different scan rates.
